# Simulation of Daily Transpiration of Tomatoes Grown in Venlo-Type Greenhouse Substrates

**DOI:** 10.3390/plants13030374

**Published:** 2024-01-26

**Authors:** Ping Yi, Xiaoman Qiang, Shengxing Liu, Yang Han, Yunfeng Li, Hao Liu, Jinglei Wang

**Affiliations:** 1 Key Laboratory of Crop Water Use and Regulation, Ministry of Agriculture and Rural Affairs, Farmland Irrigation Research Institute, Chinese Academy of Agriculture Sciences, Xinxiang 453003, China; 82101212136@caas.cn (P.Y.); qiangxiaoman@caas.cn (X.Q.); 13940585693@163.com (Y.H.); liyunfengcaas@163.com (Y.L.); 2 Graduate School of Chinese Academy of Agricultural Sciences, Beijing 100875, China; 3 School of Agricultural Sciences, Zhengzhou University, Zhengzhou 450001, China; a2837156358@163.com

**Keywords:** deficit irrigation, greenhouse, tomato, transpiration, yield, water use efficiency

## Abstract

An appropriate water supply strategy is imperative for obtaining tomatoes of a high yield and quality; the lack of one has caused resource wastage and quality deterioration. To determine the suitable irrigation amount and simulate daily transpiration under these optimal irrigation conditions, a two-year greenhouse cultivation experiment was conducted over 2022–2023. Commencing at anthesis, three distinct irrigation gradients were triggered and designated as irrigation controls with the lower limits set at 80% (T1), 70% (T2), and 60% (T3) of the substrate water-holding capacity. We determined the optimal irrigation amount by ranking the treatments using the TOPSIS method, balancing the tomato yield and quality. A segmented daily transpiration model under optimal irrigation conditions driven by crop and environmental factors was established using the Marquardt method and data from 2022, and the model was validated using data from 2023. The results indicated that T2 was the optimal irrigation amount, with the water use efficiency increased by 18.0%, but with a 10.9% decrease in yield, while the quality indices improved significantly. The *R*^2^ values of the segmented model in the flowering and fruit-setting stage and the picking stage were 0.92 and 0.86, respectively, which could provide support for optimized water management for tomato planting in greenhouse substrate cultivation.

## 1. Introduction

Tomatoes are extensively cultivated in greenhouses [1,2]. Soil-less cultivation techniques have gained widespread attention, given their advantages in overcoming rotation obstacles, increasing yields, and saving energy and labor [3,4]. Coconut coir, which is characterized by a low cost and minimal environmental risk, is an excellent substrate for soilless cultivation [5]. Irrigation substantially determines the yield and quality of substrate-cultivated tomatoes [6,7]. Using current production practices, the irrigation amount is often overlooked in favor of unilaterally increasing the yield. Apart from resulting in water and nutrient loss, excessive irrigation triggers pest and disease, leading to a decline in the tomato quality [8]. Moderate water stress, by contrast, can enhance the tomato quality and water use efficiency, while maintaining the yield [9,10]. Given these considerations, it is important to investigate the optimal irrigation strategy for substrate-grown tomatoes and establish a corresponding transpiration estimation model under substrate-cultivated conditions.

The FAO recommends using the product of reference crop transpiration (*ET*_0_) and crop coefficient (*K_c_*) to determine crop transpiration under standard conditions. This value (*ET_c_*) is then is multiplied by a specific stress index (*Ks*) to obtain the crop transpiration under stress (*ET_a_*) [11]. As the transpiration of greenhouse crops is mainly influenced by micrometeorological factors under sufficient water supply conditions [12], *ETc* can also be evaluated indirectly using greenhouse micrometeorological data [13]. Prior studies have focused on simulating transpiration under a sufficient water supply condition, such as the FAO Penman [14], FAO Radiation [15], FAO-56 Penman–Monteith [11], Hargreaves [16], and Priestley–Taylor models [17]. However, given that these indirect models are based on specific climatic conditions and the modeling parameters are difficult to obtain, their generalization and application in production practice are limited. Therefore, other researchers [18,19] tried to simplify the model based on the correlation between crop transpiration and the greenhouse microenvironment, including factors such as radiation, temperature, and humidity. For instance, some models are based on cumulative solar radiation [20,21], while others are based on cumulative pan evaporation [22,23]. These simplified models have been extensively applied in production practices due to their simplicity and practicality. However, these indirect models are based on a single variable. Regarding a particular crop, the transpiration rate depends not only on the crop’s growth and development stage, but also on other factors, such as the temperature, wind speed, and water vapor pressure deficit [24]. Therefore, other researchers performed the multivariate fitting of greenhouse meteorological factors, crop factors, and measured transpiration value and used the optimized equation obtained by multivariate fitting to estimate the rate of transpiration [25]. The transpiration rate of a crop changes throughout its growth, and the greenhouse micrometeorological factors also change. Liu et al. [26] and Li et al. [27] established multivariate nonlinear transpiration estimation models for greenhouse-grown melons and tomatoes, respectively. However, the versatility of the model may be poor as it lacks consideration for the changes in water consumption intensity and greenhouse microenvironment during crop growth and development. In addition, due to the interaction between crop transpiration and the greenhouse microenvironment, the effect of the former on the latter gradually increases with the growth and development of the crops [28]. Therefore, the sensitivity of the crop to the greenhouse microenvironment is different at different growth stages [29]. The correlation between daily transpiration and the greenhouse microenvironment at the different growth stages of crops must be explored, and a segmented daily transpiration model of the water demand of crops must be established.

Therefore, the objectives of this study were the following: (1) investigate the influence of irrigation on tomatoes and detect the optimal amount of water; (2) simulate daily transpiration under optimal irrigation conditions. We took tomatoes grown in a greenhouse substrate as the research subject and set three different irrigation gradients. The changes in the water consumption characteristics, morphological parameters, and greenhouse microenvironment of tomatoes were observed in a two-year experiment. A comprehensive evaluation of the irrigation levels was conducted based on their impact on the yield, quality, and water use efficiency to determine the optimal irrigation level. A multivariate estimation model of the daily transpiration of tomatoes at different growth stages under deficit irrigation was established, which could provide a theoretical basis for high-yield, high-quality tomato production in a Venlo-type greenhouse substrate culture.

## 2. Materials and Methods

### 2.1. Experiment Site and Plant Information

**Figure 1:**
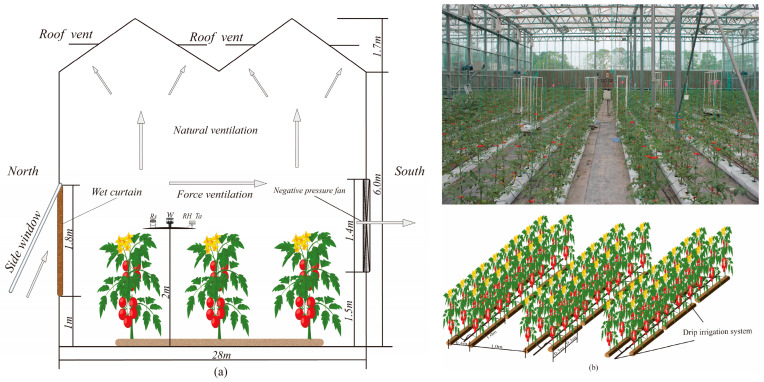
Schematic side view of the experimental greenhouse and plant information. (**a**) is the schematic side view of the experimental greenhouse, the arrow in the figure refers to direction of airflow under different ventilation conditions. (**b**) is the experiment photo and plant information, including plant spacing and row spacing.

The experimental site was located in Xinxiang City, Henan Province (35°19′ N,113°53′ E; altitude: 73.2 m). The average annual temperature is 14.1 °C, the frost-free period lasts for 210 d, and the annual sunshine duration is 2398.8 h. The experiment was carried out in a Venlo greenhouse at the Xinxiang Comprehensive Experimental Base of the Chinese Academy of Agricultural Sciences. The greenhouse was situated north, facing south. The main frame was made from light, hot-dipped galvanized steel, and the outer sheath was made from 8 mm-thick double-layer glass, covering an area of 560 m^2^ (28 m × 20 m). The specific greenhouse structure information is shown in Figure 1a.

The growth of the tomatoes was divided into three stages: the seedling, flowering and fruit-setting, and picking stages. The experimental tomato variety was *Provence*, which was planted on 21 August 2022 and 28 March 2023. The seedling stage lasted from 21 August to 27 September 2022 and from 28 March to 27 April 2023; the flowering and fruit-setting stage lasted from 27 September to 9 November 2022 and from 27 April to 31 May 2023; the picking stage lasted from 9 November 2022 to 9 January 2023 and from 31 May 2023 to 8 July 2023. Coconut coir was used as the cultivation substrate in the experiment, with a sufficient water supply at the seedling stage. During the flowering and fruit-setting stages, the water content of the substrate was taken as a control factor, and three lower limits were set, which were 60% (T1), 70% (T2), and 80% (T3) of the water-holding capacity of the substrate. Each treatment was repeated three times, and 24 plants were studied for each trial. The volume of coconut coir after foaming was 100 cm × 20 cm × 10 cm, which was wrapped in plastic film on six sides, with drainage holes only on the bottom edge. Two substrate strips were selected for each treatment, and a return tank was installed under the substrate strips. The scale (with an accuracy of 1 g) was weighed at 8:00 in the morning and 18:00 in the evening every day. The weight data in the morning and the weather conditions during the day were used as the basis for irrigation. The calculation formula is as follows [9]:

(1) $$T_{d}=W_{i}+I_{i}-R_{i}-W_{i+1}$$

where *T_d_* represents daily transpiration per tomato plant, in kg; *W_i_* represents the sum of the weight of the substrate tank and the substrate and plant weights at 8:00 on day *i*, in kg; *I_i_* represents the amount of irrigation on day *i*, in kg per plant; *R_i_* represents the irrigation return volume on day *i*, in kg; and *W_i+_*_1_ represents the sum of the substrate tank, substrate, and plant weights at 8:00 on day *i +* 1. Due to the coconut chaff being wrapped in a six-sided plastic film, the evaporation of the substrate surface was negligible. The transpiration of tomato plants grown in the substrate was measured by weighing lysimeters (accuracy = 1 g). Three tomato plants were planted in the lysimeter with the same spacing as the other plants. To minimize the boundary effects, the lysimeter was placed in the middle of the greenhouse, and records were taken every half an hour. The transpiration measurement, *T_m_*, is expressed in energy units (mm·d^−1^), which was transformed using the following formula [28]:

(2) $$T_{m}=\lambda \frac{PD\times \Delta m\times 35.3}{\Delta t}$$

where *λ* represents the latent heat of water vaporization, in w·m^2^; *PD* represents the planting density of tomato, in plants per m^2^; and Δ*m* represents the change in the quality of the substrate tank during the period Δ*t* (s), in g.

### 2.2. Measurement Items and Methods

An automatic meteorological recording system was installed at a height of 2 m in the center of the greenhouse, as shown in Figure 1a. The monitoring items included solar radiation (*Rs*), relative humidity (*RH*), air temperature (*Ta*), and wind speed (*W*). *Rs* was measured with a radiometer (LI200X, Campbell Scientific, Inc., Logan, UT, USA) with an accuracy of 0.2 kW (m^2^ (mV))^−1^. *Ta* and *RH* were measured with a temperature and humidity sensor (CS215, Campbell Scientific, Inc., Logan, UT, USA). The wind speed above the canopy was measured with an ultrasonic anemometer (Wind Sonic, Gill, London, UK) with an accuracy of 0.02 m/s. All data were recorded with intervals of 10 s, and the average was calculated once every 30 min and stored in the CR1000 data collector (Campbell Scientific Inc., Logan, UT, USA). All sensor probes were calibrated before the experiment began.

Two weeks after transplanting, six uniform plants in each treatment group were marked. The plant height, stem diameter, leaf length, and maximum leaf width of the plants were measured every 10 days, and the leaf length and width and plant height were measured with a tape measure. The leaf area index (*LAI*) is the ratio of leaf area per plant to unit area, and the leaf area per plant of tomato is the product of a single leaf’s area (leaf length × maximum leaf width) and the reduction coefficient of 0.64 [30]. The *LAI* per day within the time interval of two measurements was obtained with a piecewise cubic Hermite interpolation using MATLAB R2022a software. The stem diameter was measured at 2 cm from the stem base in two directions with a digital caliper (accuracy = 0.01 mm), which was recorded as the stem diameter. At the maturity stage the yields of 12 representative plants in the middle of each treatment plot were studied. Each treatment was repeated three times, and the number of red and pest-free fruits picked from 12 plants was recorded. The weight of each single fruit was weighed using an electronic balance with an accuracy of 0.1 g, and the total yield was calculated. The water use efficiency (*WUE*, kg·m^−3^) was calculated using the following formula [8]:

(3) $$WUE=\frac{Y_{a}}{T}$$

where *Y_a_* represents the yield of tomatoes (kg·plant^−1^) and *T* represents the water consumption of the crop (m^3^ per plant^−1^).

The crop was planted in wide and narrow rows, with a wide row spacing of 1 m, a narrow row spacing of 0.4 m, and a plant spacing of 0.3 m, as shown in Figure 1a. The nutrient solution formula was the Hoagland solution. The watering method was drip irrigation. A drip irrigation pipeline was laid in each row. The dripper flow rate was 1.98 L·h^−1^, and the dripper spacing was the same as the plant spacing. An irrigation controller and a water meter accurate to 0.001 m^3^ were installed at the head of each plot to irrigate the tomatoes regularly and strictly control the irrigation.

There were two ventilation modes in the greenhouse: natural and forced ventilation, as shown in Figure 1a. Natural ventilation was provided by the combination of a side window and roof vent. Before topping, to prevent the steep growth of seedlings, forced ventilation was controlled with a wet curtain and negative pressure fans. After topping during the picking period, forced ventilation was adopted to also provide external shade. When the temperature exceeded 30 °C, the forced ventilation system turned on. Conversely, the roof vent and side window were closed when the temperature was lower than 15 °C, and natural ventilation was applied at other times. Six mature fruits with uniform size and color and no damage were selected from each plot for measuring the quality indices. Total soluble solids were measured using a portable sugar meter digital refractometer (ATAGO, PR-32α, Tokyo, Japan). Vitamin C was measured using the 2,6-dichlorophenol indophenol sodium titration method. Soluble sugar content was determined via enthrone colorimetry. Soluble acid was measured via titration [31].

Microsoft Excel 2019 was used for statistics; SPSS 25 and MATLAB R2022a software were used to process and analyze the data. The mean absolute error (*MAE*), root-mean-square error (*RMSE*), mean relative error (*MRE*), and Nash–Sutcliffe efficiency coefficient (*NSE*) were used to evaluate the accuracy of the model simulation. The calculation formulae are as follows [13]:

(4) $$MAE=\frac{1}{n}\sum_{i=1}^{n}\left|T_{i}-M_{i}\right|$$

(5) $$RSME=\sqrt{\frac{1}{n}\sum_{i=1}^{n}{\left(T_{i}-M_{i}\right)}^{2}}$$

(6) $$MRE=\frac{1}{n}\sum_{i=1}^{n}\left(\left|\frac{T_{i}-M_{i}}{M_{i}}\right|\right)$$

(7) $$NSE=1-\frac{\sum_{i=1}^{n}\left(T_{i}-M_{i}\right)}{\sum_{i=1}^{n}\left(M_{i}-\bar{M_{i}}\right)}$$

where *n* represents the number of samples; *T_i_* represents the *i*th simulated value of the model calculation; *M_i_* represents the *i*th measured value of the lysimeter measurement; and *M* represents the average value of *M_i_*. When the *NSE* is close to 1, the model fitting effect is better.

## 3. Results

### 3.1. Variation in Microclimate Parameters and Daily Transpiration under Different Irrigation Conditions

**Figure 2:**
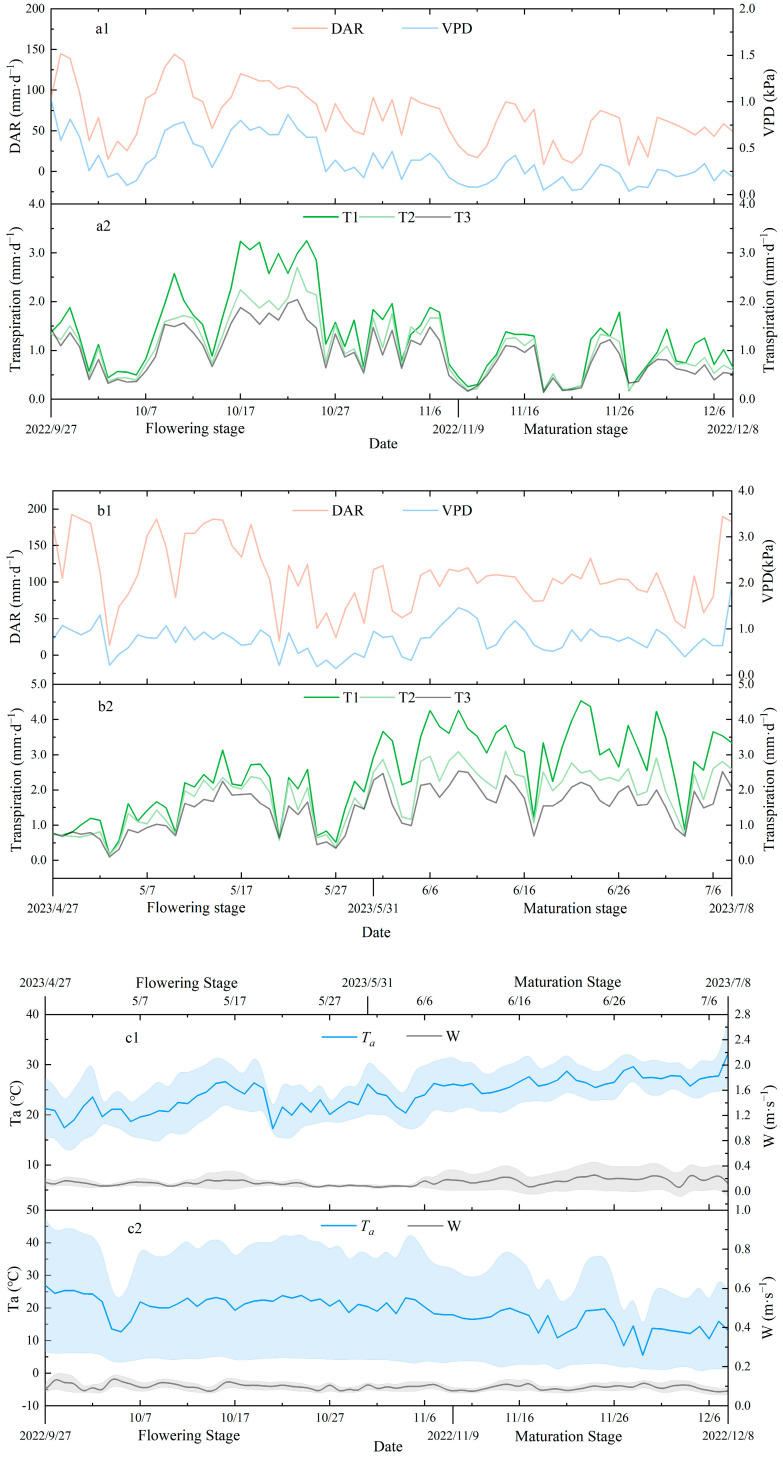
Variations in greenhouse microenvironmental factors and daily transpiration of tomatoes under different irrigation conditions in 2022 and 2023. *DAR* represents daily accumulated solar radiation, *VPD* represents daily averaged vapor deficit, *Ta* represents daily averaged air temperature, and *W* represents daily averaged wind speed. (**a2**,**b2**) are the variation of *DAR* in 2022 and 2023. (**a1**,**b1**,**c1**,**c2**) are the variation of microenvironmental factors in 2022 and 2023, the bule and grey area in (**c1**,**c2**) represents standard deviation of air temperature and wind speed recorded by the meteorological station every 30 min during a day, respectively.

Figure 2 shows the dynamic changes in daily accumulated solar radiation (*DAR*), wind speed (*W*), water vapor pressure deficit (*VPD*), air temperature (*Ta*), and daily transpiration of the tomatoes under different irrigation conditions from flowering and fruit-setting to picking in 2022 and 2023. As shown in the diagram, there are obvious differences in the *DAR*, *VPD,* and *Ta* at the different growth stages. In 2022, the daily average *DAR* values during the flowering and fruit-setting period and the picking period were 83.67 and 50.60 mm·d^−1^, respectively, while the daily average *VPD* values were 0.48 and 0.27 kPa, and the daily average *Ta* values were 21.27 and 16.06 °C, respectively. In 2023, the daily average *DAR* values were 119.70 mm·d^−1^ and 99.21 mm·d^−1^, while the daily average *VPD* values were 0.71 kPa and 0.84 kPa, and the daily average *Ta* values were 21.99 °C and 26.23 °C, respectively. The *DAR* showed a significant correlation with the *VPD* and *Ta*, and the *VPD* and *Ta* fluctuated with the fluctuation in the *DAR*. At the daily scale, *Ta* fluctuated more in 2022 than it did in 2023, as shown in Figure 2c1,c2 There was no significant difference in wind speed between 2022 and 2023, and the average *W* values in the flowering and fruit-setting periods were 0.10 and 0.12 m·s^−1^, respectively. The average wind speeds in the picking stages were 0.09 and 0.17 m·s^−1^ in 2022 and 2023, respectively. The two-year average for the maximum daily wind speed did not exceed 0.3 m·s^−1^. Notably, there was no significant difference in wind speed between the flowering and fruit-setting and picking periods in 2022, which was almost close to 0. However, in 2023, there were differences in the daily scale and wind speed changes at the different growth stages. The wind speed fluctuation during the picking period was stronger than that during the flowering and fruit-setting periods, as shown in Figure 2c2.

The variations in the daily transpiration of tomatoes cultivated under different irrigation conditions in 2022 and 2023 are shown in Figure 2a2,b2. The water demand of the tomatoes under different irrigation conditions tended to increase daily at the initial stage. The daily transpiration rate increased gradually with the increase in the *LAI* at the flowering stage. There were differences in the daily transpiration rate at the different irrigation levels. These differences were most significant when the intensity of water demand reached its peak. In 2022, the tomatoes’ water demand peak occurred during the fruit enlargement period in mid- and late October. During this peak (15–25 October), the daily average transpiration rates of T1, T2, and T3 were 2.9 mm·d^−1^, 2.1 mm·d^−1^, and 1.7 mm·d^−1^, respectively. In 2023, the maximum water demand occurred at the picking stage. During this period (5–15 June), the daily average transpiration rates of T1, T2, and T3 were 3.7 mm·d^−1^, 2.7 mm·d^−1^, and 2.1 mm·d^−1^, respectively. The daily transpiration rate remained relatively stable with the *LAI* after entering the picking period. The daily water requirements varied across the different growth stages, while the daily transpiration rate gradually decreased with the decrease in irrigation amount. The average daily transpiration rates of T1, T2, and T3 during the flowering and fruit-setting period were 1.96 mm·d^−1^, 1.53 mm·d^−1^, and 1.30 mm·d^−1^ in 2022. In contrast, during the picking period, the daily transpiration rates were 0.88 mm·d^−1^, 0.75 mm·d^−1^, and 0.62 mm·d^−1^ for T1, T2, and T3, respectively. In 2023, the average daily transpiration of T1, T2, and T3 in the flowering and fruit-setting period was 1.74 mm·d^−1^, 1.48 mm·d^−1^, and 1.24 mm·d^−1^, and the daily transpiration rates in the picking period were 3.24 mm·d^−1^, 2.29 mm·d^−1^, and 1.80 mm·d^−1^, respectively.

### 3.2. Effect of Different Water Deficits on Morphological Characteristics of Tomatoes

During growth, the physiological indices of T1, T2, and T3 increased at first, reached their maximum value, and then slightly decreased, but remained relatively stable. The trends of the growth indices of tomatoes under different irrigation conditions were basically the same, as shown in Table 1 and Table 2.

As shown in Table 1 and Table 2, the effect of a water deficit was significant for the *LAI* and plant height (*p* < 0.01), but not for the stem diameter, 50 days after transplanting (30 days after the beginning of the water deficit). At 40 days after transplanting (the 20th day of water deficit), the plant height and *LAI* of T1, T2, and T3 were significantly different (*p* < 0.05), and the plant height and *LAI* gradually decreased with the decrease in irrigation amount. A significant difference in plant height was observed among the different irrigation plots as the tomatoes grew (*p* < 0.01). The *LAIs* for T1, T2, and T3 were significantly different after 42 days, but the difference between T2 and T3 was not significant. Compared with the plant height and *LAI*, water stress had the smallest effect on the stem diameter. The differences between T1, T2, and T3 started to show after 40 and 62 days of transplanting in 2022 and 2023, respectively (*p* < 0.05).

### 3.3. Effects of Different Irrigation Amount on Yield, Quality, and Water Use Efficiency of Tomatoes

As shown in Table 3, the effect of water stress on the tomato fruit quality was significant. Apart from the total soluble solids content in 2022, the difference under different irrigation conditions reached a significant level in 2022 and 2023 (*p* < 0.01). The total soluble solids content (*TSS*), soluble sugar content (*SSC*), and organic acidity (*OA*) had a negative correlation with the irrigation level. As the irrigation amount decreased, the *TSS*, *SSC*, and *OA* levels of the fruits gradually increased, and the ratio of sugar to acid (*SAR*) also gradually increased as irrigation levels decreased. With the decrease in irrigation amount, the content of *VC* first increased, and then decreased; the levels of *VC* in the plants was ranked in the order of T2 > T3 > T1, and the content of *VC* in T2 and T3 was significantly higher than that in T1. Furthermore, the fruit nutritional quality indices were higher in 2022 than those in 2023.

As shown in Table 4, the yield of tomatoes was positively correlated with the irrigation amount, while the water use efficiency was negatively correlated with this value. The effect of a water deficit on the yield and water use efficiency (*WUE*) reached a quite significant level (*p* < 0.01). With the decrease in irrigation amount, the yield per plant gradually decreased. For example, the yield per plant of the groups T2 and T3 in 2022 decreased, respectively, by 10.94% and 21.09% compared with that of T1, and the total yield decreased by 13.17% and 23.20%. In 2023, the yield per plant of the groups T2 and T3 decreased by 13.08% and 26.15%, respectively, and the total yield reduced by 10.90% and 24.38%. With the decrease in irrigation amount compared to that of T1, the *WUE* of the groups T2 and T3 increased by 6.94% and 10.58%, respectively, in 2022; these values increased by 18.01% and 24.89%, respectively, in 2023. However, the water use efficiency of the groups T1, T2, and T3 decreased by 45.73%, 30.07%, and 27.09% more, respectively, in 2023 compared to those in 2022.

### 3.4. The Determination of Optimal Irrigation Amount Using TOPSIS

By conducting a comprehensive comparison using *TOPSIS*, *GRA,* and *PCA*, Li et al. [31] proposed that *TOPSIS* can be better applied to the comprehensive evaluation of greenhouse tomato yield and quality in north China. Based on the experimental results from 2022 and 2023, this study utilized the *TOPSIS* method and chose the yield, water use efficiency, soluble solids, VC content, and sugar–acid ratio as indicators to evaluate the different irrigation amounts. Comprehensive scores were obtained and ranked, as shown in Table 5. According to Table 5, the experiments in 2022 and 2023 had a comprehensive ranking of the irrigation methods in the order of T2 > T3 > T1. T2 had the highest comprehensive score, and T1 had the lowest one. Thus, under the conditions of high yield and high quality, the optimal irrigation amount was that of T2.

### 3.5. Modeling the Diurnal Transpiration of Tomatoes in Substrate Cultivation

#### 3.5.1. Determination of Model Factors

Based on the daily transpiration data of tomatoes measured with a lysimeter (*Tm*) in the flowering and fruit-setting and picking periods of T2 in 2022, the daily accumulative solar radiation (DAR), daily average wind speed (W), daily average water vapor pressure deficit (VPD), daily average air temperature (Ta) at a height of 2 m from the ground, and leaf area index (LAI) were selected for path and correlation analyses, as shown in Table 6.

As shown in Table 6, there were different correlations between the *Tm* and various variables at the different growth stages. The correlations between the *Tm* and other variables during the flowering and fruit-setting stages were ranked from strong to weak as follows: *VPD* > *DAR* > *LAI* > *Ta* > *W*. Here, the correlations between the *VPD*, *DAR*, *LAI*, *Ta*, and *Tm* were significant. The correlation between the *VPD* and *Tm* was the strongest, with a correlation coefficient of 0.864. The path analysis of *Tm* and the environmental factors showed that *VPD* had the biggest impact on the *Tm*, regardless of whether it was direct or indirect. However, the correlation analysis between *Tm* and the related variables in the picking period showed that the correlation between the *Tm* and *DAR* was the strongest, and the correlation coefficient between each related variable and the Tm was ranked in the order of *DAR* > *VPD* > *W* > *Ta* > *LAI*. Among them, the correlations between the *DAR*, *VPD*, *W*, *Ta,* and *Tm* reached a significant level, while the correlation between *LAI* and *Tm* was the weakest and was not significant. Meanwhile, the path analysis of *Tm* and the related variables showed that the influence of the *VPD* and *LAI* on Tm decreased, the influence of the *DAR* and *W* on *Tm* increased, and the *DAR* had the greatest influence on *Tm* both directly and indirectly.

#### 3.5.2. Establishment of Daily Transpiration Model

The analysis in Section 3.5.1 showed that the relationship between the daily transpiration rate (*Tm*) and the environmental factors changed with the growth and development of the plants. The leaf area index (*LAI*) gradually increased with crop growth and reached its peak at the end of the flowering and fruit-setting period. In this stage, the daily averaged water vapor pressure deficit (*VPD*) had the most significant effect on the Tm, followed by the daily cumulative solar radiation (*DAR*) and the *LAI*, and the correlation between the daily mean temperature (*Ta*) and the *Tm* was also significant, but the correlation between the *Tm* and wind speed (*W*) was not significant. Therefore, the *VPD*, *DAR*, *Ta,* and *LAI* were selected as parameters to simulate the *Tm* during the flowering and fruit-setting period. However, once in the picking stage, the *DAR* had the most significant effect on the *Tm*, followed by the *VPD*, *W*, and *Ta*. The correlation between the *LAI* and *Tm* was negative and not significant, which indicates that the *LAI* had a smaller effect on the *Tm*, and the *Tm* was mainly controlled by meteorological factors at this stage. Therefore, the *DAR*, *VPD*, *Ta*, and *W* were selected as parameters to simulate *Tm* during the maturation period. Based on the relationship between the chosen parameters and the *Tm* at various growth stages, Marquardt parameter estimation was utilized to derive the daily transpiration model for T2 at the different growth stages as follows:

(8) $$Ts=\left\{\begin{array}{l} 0.001DAR^{1.261}+0.689\ln\left(VPD\right)+0.399\ln\left(Ta\right)+0.527\ln\left(LAI\right)\begin{array}{cc} & 1\leq LAI<LAI_{max} \end{array}\\ 0.011DAR^{1.04}+0.189\exp(VPD)-0.110\ln\left(Ta\right)+5.625\ln\left(W\right)-6.01\begin{array}{cc} & LAI=LAI_{max} \end{array} \end{array}\right.$$

where *Ts* represents the simulated daily transpiration rate, in mm·d^−1^; *DAR* represents the daily accumulated solar radiation, in mm·d^−1^; *Ta* represents the daily average temperature; *W* represents the daily average wind speed, in m·s^−1^; and *LAI* represents the leaf area index, in m^2^·m^−2^.

### 3.6. Model Validation

Liu et al. [26] and Li et al. [27] proposed an unsegmented multivariate fitting equation for estimating the *Tm* over the full life span of the tomatoes based on the relationship between daily transpiration, *Tm*, meteorological factors, and the *LAI*, which ignored the variations in crop development and greenhouse meteorological conditions. To validate the necessity of establishing a segmented model, an unsegmented daily transpiration estimation model *T*′ was established by using the meteorological data of the measured daily transpiration rate and greenhouse for the year 2022, as shown below:

(9) T′=0.514ln(DAR)+0.08ln(VPD)+0.379ln(Ta)+1.05ln(W)+0.006exp(LAI)+0.249

where *Ts* represents the simulated daily transpiration rate, in mm·d^−1^; *DAR* represents the daily accumulated solar radiation, in mm·d^−1^; *Ta* represents the daily average temperature, in °C; *W* represents the daily average wind speed, in m·s^−1^; and *LAI* represents the leaf area index, in m^2^·m^−2^.

**Figure 3:**
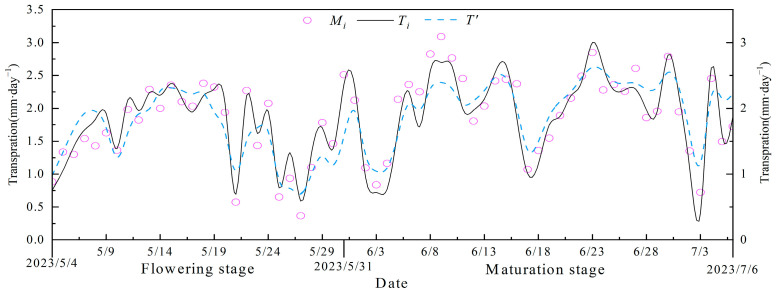
Comparison of simulated and measured values of daily transpiration; *T_i_* represents the value simulated using the segmented model, *T*′ represents the value simulated using the unsegmented model, and *M_i_* represents the measured value.

**Figure 4:**
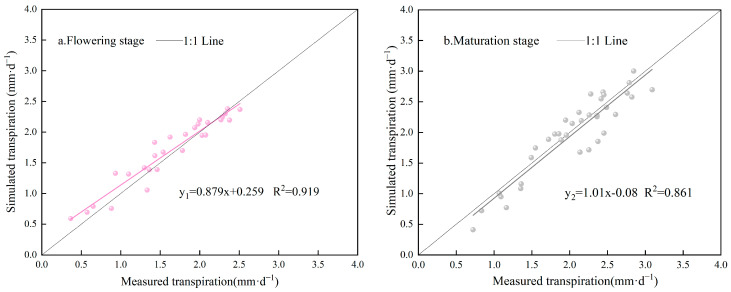
Validation of the accuracies of daily transpiration models for different fertility periods.

Figure 3 is the comparison between the daily measured and simulated values obtained by segmented modeling *Ts* and unsegmented modeling *T*′ in 2023. Table 7 depicts the comparison of the simulation accuracies of the *Ts* and *T*′ in the flowering and fruit-setting and picking stages. As shown in Figure 3 and Table 7, unsegmented modeling *T*′ underestimated the *Tm* at the flowering and fruit-setting stage, with a mean absolute error (*MAE*) and root-mean-square error (*RSME*) of 0.27 mm·d^−1^ and 0.34 mm·d^−1^, respectively, and an *NSE* of 0.66. The mean relative error *MRE* of the segmented model *Ts* was improved by 5% compared with that of the *T*′ model, and the *MAE, RSME,* and *NSE* were 0.15 mm·d^−1^, 0.18 mm·d^−1^, and 0.91, respectively, which were smaller than those of the *T*′ model. The simulation accuracies of the *T*′ model were improved during the maturation period, at 12.99%, 0.25 mm·d^−1^, and 0.30 mm·d^−1^ for the *MRE*, *MAE,* and *RSME*, respectively, while those of the *Ts* model were 11.07%, 0.20 mm·d^−1^, and 0.25 mm·d^−1^. In addition, the *NSE* of the *Ts* model simulation is 0.85, while the *NSE* of the *T*′ model is 0.73; thus, the *Ts* model was still better than the *T*′ model. Figure 4 shows the linear fit of the simulated and measured values of the segmented model *Ts* at the flowering and maturation stages. The correlation coefficients *R*^2^ of the *Ts* model at the flowering and maturation stages were 0.92 and 0.86, respectively. The synthesis of the statistical indicators in Table 7 and Figure 3 and Figure 4 shows that the simulation accuracy of the *Ts* model was higher than that of the *T*′ model; in addition, fewer parameters were required by the *Ts* model than by the *T*′ model. Therefore, the segmented model *Ts* was better than the unsegmented model *T*′, which could accurately simulate the daily transpiration of Venlo-type greenhouse substrate-cultivated tomatoes.

## 4. Discussion

The aim of facility vegetable production has always been to achieve a high yield and quality [8,31]. Several studies [32,33,34] have indicated that an appropriate degree of water stress can improve the quality of tomatoes, while ensuring a stable yield. Using the current production practices, an improper water supply has resulted in wasted water resources and a reduced fruit quality [35]. To determine the optimal irrigation lower limit, this study established three different irrigation gradients (T1, T2, and T3) according to the matrix water-holding capacity from the beginning of flowering and investigated the effect of the irrigation amount on the tomato yield and quality. Previous studies [36,37] have shown that crop transpiration is positively correlated with the irrigation amount, and crop quality is negatively correlated with irrigation amount within a certain threshold. In this study, with the increase in irrigation amount, the daily transpiration rate, plant height, stem diameter, *LAI*, and yield increased. The water use efficiency (*WUE*), total soluble solids (*TSS*), soluble sugar content (*SSC*), and sugar–acid ratio (*SAR*) decreased progressively with the increase in irrigation amount. The *VC* content increased first, and then decreased with the decreased irrigation amount (Table 1, Table 2, Table 3 and Table 4), which is consistent with the results of previous studies [8,31,37]. However, compared with other studies in a nearby area [8,24,31], it was found that under the same irrigation conditions, the quality indices, such as the *TSS*, *SAR*, and *SSC*, of the tomatoes in this study were better than those of the -soil grown tomatoes, which is consistent with the results of several studies [38,39,40]. Due to the different effects of a water deficit on the different yield and quality indicators, the comprehensive evaluation of different irrigation amounts in this study was conducted by utilizing the *TOPISIS* method. The results showed that the T2 treatment with a 70% matrix water-holding capacity significantly improved the *WUE* and quality of the tomatoes when there was no significant decrease in yield, and the comprehensive score was the highest (Table 5). Therefore, it is recommended to take a 70% matrix water-holding capacity as the lower limit of irrigation for high-yield and high-quality tomato production. However, due to the different sensitivities of tomatoes to water at the different growth stages [41], the impact of water stress on the tomato yield and quality during the various growth stages remains to be elucidated. Therefore, different water treatments need to be conducted at different growth stages to further investigate the effects of water stress on the yield and quality of tomatoes grown in a substrate.

In this study, correlation and path analyses were conducted on the daily transpiration rate (*Tm*) and environmental factors in the flowering, fruit formation, and maturation periods of tomatoes grown in a substrate (Table 6). The results indicated that the *Tm* at the different growth stages was mainly influenced by meteorological factors. During the flowering and fruit formation stage, the *Tm* was primarily influenced by the vapor pressure deficit (*VPD*), while during the picking period, the *Tm* was primarily influenced by the daily cumulative amount of solar radiation (*DAR*). This may be attributed to the interaction between the greenhouse microenvironment and plants [42]. Crop transpiration causes cooling and humidification [30], and the transpiration rate of crops will gradually increase during growth. The canopy of the greenhouse may obstruct ventilation, which will increase during crop growth, resulting in a gradual increase in the volatility of the wind speed (Figure 2c2). Additionally, as the crops grew in 2023, the temperature gradually increased due to external environment influences, and the relationship between the *Tm* and meteorological factors, such as wind speed and temperature, also changed. For instance, previous studies [43,44] have shown that a temperature of 25 °C is the most suitable for tomato growth and development. In the range from 15 °C to 25 °C, the increase in temperature promotes the growth and development of tomatoes. When the temperature exceeds 25 °C, the growth and development of tomatoes are inhibited [45]. In 2023, the average daily temperature during the tomato flowering and fruit-setting period was 21.99 °C, while it was 26.23 °C during the picking period. The increased temperature during the flowering and fruit formation stage promoted the growth of the tomatoes. However, as they grew, the *Ta* gradually increased, and the increase in temperature during the picking period inhibited the growth of the tomatoes. The impacts of temperature on the daily transpiration of tomatoes in a substrate culture were different at the flowering and fruit-setting and picking periods. Due to the selection of model parameters, the *Tm* gradually increased with the increase in *LAI* during the flowering and fruit-setting period, and the correlation between the daily transpiration rate of tomatoes and the *LAI* reached a significant level (*p* < 0.01). To accurately simulate the *Tm*, the change in *LAI* must be accounted for, while considering the meteorological factors. During the picking period, the *LAI* remained stable, and the correlation between the *Tm* and *LAI* was not significant. The *Tm* fluctuated with the *DAR*, and the *Tm* was influenced by meteorological factors. Thus, it was imperative to adjust the model parameters to match the crop’s growth period alterations.

The *FAO* indicated that the estimation of evapotranspiration based on pan-evaporation data for a period of 10 days or longer was warranted [11]; however, the water-holding capacity of the substrate was much lower than that of the soil. Thus, this method, which is widely used for the irrigation management of greenhouse soil-cultivated crops, could not satisfy the requirement of precise irrigation management in greenhouses. The Penman–Monteith model, which is the most widely used approach in evapotranspiration simulation [15,46], has been validated under various climatic conditions around the world; yet, it is difficult to obtain the necessary parameters, limiting its application in practical production [29]. Therefore, when simulating the daily transpiration of tomatoes cultivated in a greenhouse substrate, instead of following the lead of the previous studies [46,47], which first determined the potential transpiration of the crop and then determined the daily transpiration under insufficient irrigation conditions by calculating the stress index, this study screened the model parameters by analyzing the relationship between the daily transpiration of tomatoes under deficient irrigation conditions and the factors affecting daily transpiration (Table 6). A segmented model for the daily transpiration of tomatoes grown in a Venlo greenhouse substrate was established based on the correlation between daily transpiration and environmental factors at the different growth stages, where the greenhouse environmental factors and *LAI* were the driving forces, the experimental data from 2022 were used for training, and the experimental data from 2023 were used for verification. The *R*^2^ during the flowering and fruit-setting and picking periods were 0.92 and 0.86, respectively (Figure 3 and Figure 4). The segmented model proposed in this study, compared with the model proposed by Liu et al. [26], integrates the physiological processes of the crop itself and the changes in environmental factors and expands the wind speed and water vapor pressure deficit terms, which covers the factors more comprehensively. Therefore, this model is more sensitive to the changes in meteorological parameters and more versatile. However, the training of the model was based on the 2022 fall data and verification was based on the 2023 spring data, which only verifies the applicability of the model for tomatoes grown in spring. The applicability of the model to tomatoes grown in fall must be investigated. Nevertheless, because the model parameters were easy to obtain, this is a simple and fast method for estimating the daily transpiration of tomatoes, which can serve as a basis for the irrigation of high-yield and -quality tomato production in a Venlo greenhouse in northern China.

## 5. Conclusions

Substrate cultivation can be used to more successfully obtain high-quality fruits when compared to soil cultivation. It is recommended to use a 70% substrate water-holding capacity as the lower limit of irrigation for Venlo-type substrate tomato cultivation in north China, which can increase the water use efficiency by 18.00%, while the tomato yield decreases by 10.90%. Meanwhile, the sugar–acid ratio, VC content, total soluble solids, soluble sugar content, organic acidity, and other nutritional quality of tomatoes were improved, which reflects an improved quality, while ensuring a high tomato yield. Daily transpiration during the flowering and fruit-setting stage was mainly influenced by the vapor pressure deficit, and it mostly correlated with the daily accumulated solar radiation value; the model considered the greenhouse environmental and crop factors as drivers. The *R*^2^ during the flowering and fruit-setting and picking stages was more than 0.85, which could be used to easily and effectively estimate the daily transpiration of tomatoes and could provide support for the irrigation management of high-quality and -yield tomatoes in Venlo greenhouse substrate cultivation.

## Figures and Tables

**Table 1:** The effects of irrigation amount on tomato growth indices 2022.

Growth Indices	Treatments	Days after Transplant/(d^−1^)					
		32	42	52	62	72	87
Leaf area index	T1	0.43 ± 0.08 a	0.94 ± 0.32 a	2.30 ± 0.51 a	3.54 ± 0.47 a	3.69 ± 0.93 a	3.51 ± 0.65 a
	T2	0.54 ± 0.20 a	0.85 ± 0.10 ab	1.50 ± 0.19 b	2.36 ± 0.32 b	2.64 ± 0.27 b	2.53 ± 0.34 b
	T3	0.41 ± 0.07 a	0.61 ± 0.09 b	1.23 ± 0.13 b	2.00 ± 0.19 b	2.36 ± 0.19 b	2.31 ± 0.25 b
Plant height (cm)	T1	54.78 ± 1.40 a	81.60 ± 1.23 a	133.9 ± 1.65 a	142.8 ± 1.0 a	141.8 ± 1.0 a	140.1 ± 2.5 a
	T2	54.03 ± 1.70 a	74.80 ± 1.14 b	116.2 ± 0.60 b	125.6 ± 1.4 b	127.0 ± 1.8 b	129.8 ± 1.9 b
	T3	55.08 ± 1.23 a	68.17 ± 1.53 c	104.6 ± 1.82 c	120.5 ± 1.8 c	122.0 ± 1.3 c	122.2 ± 1.1 c
Stem thickness (mm)	T1	9.49 ± 0.29 a	10.33 ± 0.18 a	12.10 ± 0.21 a	12.54 ± 0.59 a	12.78 ± 0.21 a	12.95 ± 0.68 a
	T2	9.24 ± 0.55 a	10.31 ± 0.04 a	11.61 ± 0.70 ab	11.95 ± 0.34 ab	12.19 ± 0.09 b	12.30 ± 0.11 ab
	T3	9.37 ± 0.64 a	10.22 ± 0.36 a	11.01 ± 0.30 b	11.28 ± 0.59 b	11.54 ± 0.25 c	11.77 ± 0.33 b
Probability of significance	Leaf area index	ns	*	**	**	**	*
	Plant height	ns	**	**	**	**	**
	Stem thickness	ns	ns	ns	*	*	*

The letter(s) at the end of the numbers represent significant differences at *p* < 0.05; ns means insignificant; * means significant at *p* < 0.05; ** means significant at *p* < 0.01 using the Tukey’s multiple range test.

**Table 2:** The effects of irrigation amount on tomato growth indices in 2023.

Growth Indices	Treatments	Days after Transplant/(d^−1^)					
		30	40	50	60	70	80
Leaf Area Index	T1	0.53 ± 0.07 a	1.00 ± 0.05 a	2.07 ± 0.21 a	3.27 ± 0.13 a	3.58 ± 0.45 a	3.65 ± 0.40 a
	T2	0.56 ± 0.10 a	0.86 ± 0.02 b	1.44 ± 0.08 b	2.19 ± 0.16 b	2.57 ± 0.41 b	2.64 ± 0.21 b
	T3	0.61 ± 0.13 a	0.85 ± 0.04 b	1.29 ± 0.09 b	1.99 ± 0.22 b	2.33 ± 0.34 b	2.39 ± 0.43 b
Plant height (cm)	T1	54.63 ± 1.95 a	86.23 ± 1.37 a	128.67 ± 1.61 a	136.67 ± 1.53 a	139.67 ± 1.53 a	140.50 ± 1.80 a
	T2	54.67 ± 1.04 a	81.67 ± 1.53 b	103.50 ± 1.08 b	115.38 ± 1.11 b	118.00 ± 1.32 b	126.17 ± 1.26 b
	T3	56.00 ± 1.29 a	77.95 ± 1.01 c	99.88 ± 1.03 c	110.80 ± 1.59 c	113.17 ± 0.76 c	119.67 ± 1.15 c
Stem thickness (mm)	T1	8.89 ± 0.60 a	9.31 ± 0.30 a	10.00 ± 0.20 a	12.24 ± 0.55 a	12.65 ± 0.68 a	12.67 ± 0.90 a
	T2	8.77 ± 0.57 a	9.39 ± 0.32 a	10.56 ± 0.23 b	11.18 ± 0.37 b	11.74 ± 0.73 ab	11.81 ± 0.56 ab
	T3	8.81 ± 0.57 a	9.10 ± 0.36 a	10.21 ± 0.39 b	10.97 ± 0.59 b	11.04 ± 0.61 b	11.41 ± 0.37 a
Probability of significance	Leaf Area Index	**	**	**	**	**	**
	Plant height	**	**	**	**	**	**
	Stem thickness	ns	*	*	*	*	*

The letter(s) at the end of the numbers represent significant differences at *p* < 0.05; ns means insignificant; * means significant at *p* < 0.05; ** means significant at *p* < 0.01 using the Tukey’s multiple range test.

**Table 3:** The effects of irrigation amount on fruit quality.

Year	Treatments	*TSS*/(%)	*VC*/(mg·kg^−1^)	*OA*/(%)	*SSC*/(mg·g^−1^)	*SAR*
2022	T1	5.27 ± 0.85 b	154.53 ± 5.75 b	0.43 ± 0.01 b	23.19 ± 1.01 b	4.67 ± 0.16 b
	T2	5.89 ± 0.29 ab	183.60 ± 7.07 a	0.48 ± 0.02 a	29.58 ± 1.65 a	6.15 ± 0.77 a
	T3	6.60 ± 0.20 a	172.42 ± 9.36 a	0.50 ± 0.02 a	30.42 ± 4.27 a	6.51 ± 0.93 a
Probability of significance		*	**	**	**	**
2023	T1	4.64 ± 0.15 b	130.70 ± 9.75 b	0.48 ± 0.03 b	21.02 ± 1.04 b	4.18 ± 0.07 b
	T2	5.25 ± 0.20 a	153.88 ± 10.60 a	0.54 ± 0.02 a	25.33 ± 1.66 a	4.80 ± 0.29 a
	T3	5.45 ± 0.40 a	154.67 ± 8.04 a	0.55 ± 0.04 a	26.07 ± 2.48 a	4.89 ± 0.17 a
Probability of significance		**	**	**	**	**

*TSS*: total soluble solids content; *VC*: Vitamin C; *OA*: organic acidity; *SSC*: soluble sugar content; *SAR*: sugar–acid ratio. The letter(s) at the end of the numbers represent significant differences at *p* < 0.05; * means significant at *p* < 0.05; ** means significant at *p* < 0.01 using the Tukey’s multiple range test.

**Table 4:** The effects of irrigation amount on yield and water use efficiency.

Years	Treatments	Yield per Plant (kg·plant^−1^)	Yield (t·hm^−2^)	Water Use Efficiency (kg·m^−3^)
	T1	1.28 ± 0.05 a	56.50 ± 0.87 a	38.18 ± 1.05 b
2022	T2	1.14 ± 0.01 b	49.06 ± 0.26 b	40.83 ± 0.21 a
	T3	1.01 ± 0.02 c	43.39 ± 0.91 c	42.22 ± 0.87 a
Probability of significance		**	**	**
	T1	1.30 ± 0.08 a	54.11 ± 0.99 a	26.60 ± 1.59 b
2023	T2	1.13 ± 0.06 b	48.21 ± 1.05 b	31.39 ± 1.67 a
	T3	0.96 ± 0.06 c	40.92 ± 0.67 c	33.22 ± 2.02 a
Probability of significance		**	**	**

The letter(s) at the end of the numbers represent significant differences at *p* < 0.05; ** means significant at *p* < 0.01 using the Tukey’s multiple range test.

**Table 5:** Integrated evaluation of different irrigation amounts using *TOPSIS*.

Years	Treatments	*D+*	*D−*	*Ci*	Rank
	T1	0.8844	0.4667	0.3454	3
2022	T2	0.4008	0.6942	0.6340	1
	T3	0.4966	0.8131	0.6209	2
	T1	0.8866	0.4624	0.3428	3
2023	T2	0.2706	0.7821	0.7429	1
	T3	0.4624	0.8866	0.6572	2

*D+* and *D−* represent positive and negative Euclidean distances, respectively; *Ci* represents comprehensive evaluation index.

**Table 6:** Correlation and pathway analyses between T2 daily transpiration and environmental factors at different fertility stages.

Stage	Variable	Direct Path Coefficient	Indirect Coefficient						Correlation Coefficient
			*DAR*	*W*	*VPD*	*Ta*	*LAI*	*∑*	
	*DAR*	0.057		0.022	0.051	0.020	0.006	0.098	0.795 **
	*W*	0.144	0.054		0.051	−0.068	−0.039	−0.002	0.255
Flowering	*VPD*	0.810	0.723	0.287		0.360	0.031	1.400	0.864 **
	*Ta*	0.087	0.030	−0.041	0.039		0.005	0.033	0.384 *
	*LAI*	0.414	0.044	−0.113	0.016	0.025		−0.028	0.405 **
	*DAR*	0.738		0.265	0.590	0.401	−0.179	1.077	0.888 **
	*W*	0.246	0.088		0.057	0.022	0.075	0.243	0.520 **
Maturation	*VPD*	0.085	0.068	0.020		0.034	−0.041	0.081	0.583 **
	*Ta*	0.025	0.014	0.002	0.010		0.009	0.035	0.421 **
	*LAI*	0.029	−0.007	0.009	−0.014	0.011		−0.001	−0.183

*DAR*, daily accumulated solar radiation, in mm·d^−1^; *W*, daily average wind speed, in m·s^−1^; *VPD*, vapor pressure deficit, in kPa; *Ta*, daily average air temperature, in °C; *LAI*, leaf area index, in m^2^·m^2^. * means significant at *p* < 0.05; ** means significant at *p* < 0.01.

**Table 7:** Comparison of simulation accuracies of *T*′ and *Ts* models.

Stage	Model	Statistical Index			
		*MRE*	*MAE*	*RSME*	*NSE*
Flowering	*T*′	17.66	0.27	0.34	0.66
	*Ts*	12.63	0.15	0.18	0.91
Maturation	*T*′	12.99	0.25	0.30	0.73
	*Ts*	11.07	0.20	0.25	0.83

*MRE*, mean relative error; *MAE*, mean absolute error; *RSME*, root-mean-square error; *NSE*, Nash–Sutcliffe efficiency coefficient.

## Data Availability

The raw data supporting the conclusions of this article will be made available by the authors, without undue reservation. The data are not publicly available due to copyrights cannot be available.
